# High‐Resolution 3D Printing of Freeform, Transparent Displays in Ambient Air

**DOI:** 10.1002/advs.201901603

**Published:** 2019-10-04

**Authors:** Hyeon Seok An, Young‐Geun Park, Kukjoo Kim, Yun Seok Nam, Myoung Hoon Song, Jang‐Ung Park

**Affiliations:** ^1^ Department of Materials Science and Engineering Nano Science Technology Institute Yonsei University Seoul 03722 Republic of Korea; ^2^ Center for Nanomedicine Institute for Basic Science (IBS) Yonsei‐IBS Institute Seoul 03722 Republic of Korea; ^3^ Electronics and Telecommunications Research Institute (ETRI) Daejeon 34129 Republic of Korea; ^4^ School of Materials Science and Engineering Ulsan National Institute of Science and Technology (UNIST) Ulsan 44919 Republic of Korea

**Keywords:** 3D printing, optoelectronics, printable electronics, transparent displays

## Abstract

Direct 3D printing technologies to produce 3D optoelectronic architectures have been explored extensively over the last several years. Although commercially available 3D printing techniques are useful for many applications, their limits in printable materials, printing resolutions, or processing temperatures are significant challenges for structural optoelectronics in achieving fully 3D‐printed devices on 3D mechanical frames. Herein, the production of active optoelectronic devices with various form factors using a hybrid 3D printing process in ambient air is reported. This hybrid 3D printing system, which combines digital light processing for printing 3D mechanical architectures and a successive electrohydrodynamic jet for directly printing transparent pixels of organic light‐emitting diodes at room temperature, can create high‐resolution, transparent displays embedded inside arbitrarily shaped, 3D architectures in air. Also, the demonstration of a 3D‐printed, eyeglass‐type display for a wireless, augmented reality system is an example of another application. These results represent substantial progress in the development of next‐generation, freeform optoelectronics.

## Graphical abstract

A hybrid, 3D printing system based on digital light processing and an electrohydrodynamic inkjet is developed for producing freeform optoelectronic devices in ambient air. This 3D printing system can print all components of the device as well as the mechanical frames; thus, the system can produce transparent 3D architectures with arbitrary shapes at the desired locations.

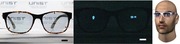

The next generation of optoelectronics with unique 3D architectures has been explored extensively over the past decade as the key to moving beyond the inherent planarity associated with conventional, photolithography‐based microfabrication techniques.^1^, ^2^, ^3^ However, these lithography‐based processes require multiple fabrication steps, including vacuum depositions or etchings that have high fabrication costs and cause unnecessary waste of materials. In addition, the photolithography is only for 2D structures on a planar surface. These complicated, 2D‐based processing steps limit the application for next‐generation electronics that have arbitrary, 3D structures. Thus, the optoelectronic devices with 3D structures need to be fabricated via stacks of 2D device layers, in which interconnects between layers are achieved using wire‐bonding or a metal vial,^4^ but the use of these layer‐by‐layer stacking approaches can limit the geometries of devices that have more complex 3D shapes. Overcoming this limitation potentially could result in significant applications beyond improving the scalability in device integration technologies. For instance, the ability to seamlessly incorporate optoelectronics into 3D structures could impart enhanced functionalities to these devices, such as advanced optical, electrical, and mechanical properties.^5^, ^6^, ^7^, ^8^, ^9^

One of the alternative technologies that can overcome the limitations of photolithography is direct 3D printing, which has been explored extensively during the last several years for purely additive operations in which functional inks are deposited only where they are required for the 3D structures. For example, printing technologies, such as direct ink writing,^10^, ^11^, ^12^, ^13^, ^14^ digital light processing (DLP), selective laser sintering (SLS),^15^ fused deposition modeling (FDM),^16^ and inkjet printing,^17^ can be adapted to print 3D structures for a wide range of applications including electronic circuits,^18^ batteries,^19^ photonic structures, and optoelectronic devices.^20^, ^21^, ^22^, ^23^, ^24^ However, multi‐functional inks, such as semiconductors, dielectric layers, electrodes, and light‐emitting materials, cannot be printed using FDM, SLS, or DLP methods, but only plastic or metal‐based 3D structures are achievable. Also, the resolutions of these conventional 3D printing techniques typically are limited to a scale greater than approximately 30 µm. Although this printing resolution is enough for the lighting devices, these 30 µm patterns can be too large for recent high‐definition displays.^25^ Also, the 3D assembly technologies have been used for forming 3D structures with precise structural programming.^22^, ^26^, ^27^, ^28^, ^29^

Herein, we report a hybrid 3D printing system that combines DLP and electrohydrodynamic jet (e‐jet) printing for the production of transparent and freeform 3D optoelectronic devices in ambient air (Figure S1a, Supporting Information). The DLP method can print transparent plastic frames with arbitrary 3D shapes. The 3D e‐jet printing method is used successively for direct printing of high‐resolution organic light‐emitting diode (OLED) pixels onto these DLP‐printed, 3D mechanical frames. This 3D e‐jet can print diverse functional materials for optoelectronic devices with high resolutions (minimum linewidth: 2.6 µm), even on 3D architectures with diverse geometries, by adjusting the printing speed and position within a synchronized, five‐axis stage movement. This hybrid 3D system can print all of the components of optoelectronic devices in series, from 3D mechanical frames to all OLED layers, including encapsulations, as a transparent, freeform 3D display. Also, the processing steps of this hybrid 3D printing system require no additional thermal drying/annealing steps, which enables the direct formation of 3D devices with high resolutions at ambient conditions. Although previous approaches on flexible/stretchable devices required the use of substrates and, therefore, must be attached only onto the surface areas of nonplanar objects,^9^, ^30^, ^31^, ^32^, ^33^, ^34^, ^35^, ^36^, ^37^, ^38^, ^39^, ^40^ this hybrid 3D printing system can embed optoelectronic devices inside arbitrarily shaped 3D architectures selectively at desired locations instead of the surface regions, because all of the devices and mechanical frames are printed together as freeform optoelectronics. Demonstrations of various 3D architectures, which include high‐resolution OLED pixels inside the architectures and a transparent, eyeglass‐type display for a wireless, augmented reality system, provide examples of the applications of this hybrid 3D printing system and indicate the future promise of freeform 3D optoelectronics.

**Figure** **1**a illustrates the hybrid 3D printing system (based on DLP and e‐jet) that can be used to form freeform, transparent OLED arrays in ambient air. This hybrid 3D printing system can print all components of transparent OLEDs as well as 3D mechanical frames, which indicates that it can achieve multi‐material integrations for embedding active optoelectronic devices inside 3D architectures that have arbitrary shapes. Figure 1b,c illustrates the printing process and the layouts of these freeform, transparent OLED arrays. The printing steps are configured to print individual layers of different functional materials, including plastics for 3D mechanical architectures, each layer for transparent OLEDs, and the encapsulation layer. This printing process consists of three steps, i.e., 1) polylactide (PLA) or transparent polycarbonate (PC) is printed with arbitrary 3D shapes using a commercially available DLP printer (Ackuray A96, ACKURETTA); 2) subsequently, the pixel arrays of transparent OLEDs are printed on the DLP‐printed 3D surface using a homemade e‐jet printing system; and 3) the top surface of the OLED arrays is encapsulated using the DLP printer. These OLEDs printed on the 3D architectures are designed to have the following layers, i.e., 1) silver nanowires (AgNWs, B 424‐1, Nanopyxis) with an average diameter of 23 nm and length of 27 µm are electrosprayed to form their random networks as the bottom transparent electrode (sheet resistance: 14.12 Ω sq^−1^; transmittance: 92% in the visible light range) of OLEDs on the surface of the 3D architecture; 2) photocurable polyurethane (PU, NOA 74, Norland Products), which is printed as the pixel defining layer (PDL, thickness: 194 ± 4 nm) using the e‐jet method before UV curing; 3) poly(ethylenedioxythiophene):polystyrene sulfonate (PEDOT:PSS, Clevios AI 4083, Heraeus) is printed as the hole transport layer (HTL, thickness: 94 ± 4 nm) using the e‐jet method; 4) 4,4′‐bis[4‐(di‐*p*‐tolylamino)styryl]biphenyl‐doped 2‐*tert*‐butyl‐9,10‐di(naphtha‐2‐yl)anthracene (TBADN:DPAVBi, thickness: 48 ± 2 nm) (Lumtec) or SPW‐111 (a white polymer known as Merck White, Merck, thickness: 53 ± 3 nm) is e‐jet printed as the light emission layer (EML); 5) poly[(9,9‐bis(3′‐(*N*,*N*‐dimethylamino)propyl)‐2,7‐fluorene)‐alt‐2,7‐(9,9‐dioctylfluorene)] (PFN, LT‐N4027, Lumtec) is electro‐sprayed as the electron transfer layer (ETL, thickness: 41 ± 2 nm), 6) random networks of AgNWs were electro‐sprayed by an e‐jet as the top transparent electrode before printing the encapsulation layer of the transparent PC.

**Figure 1:**
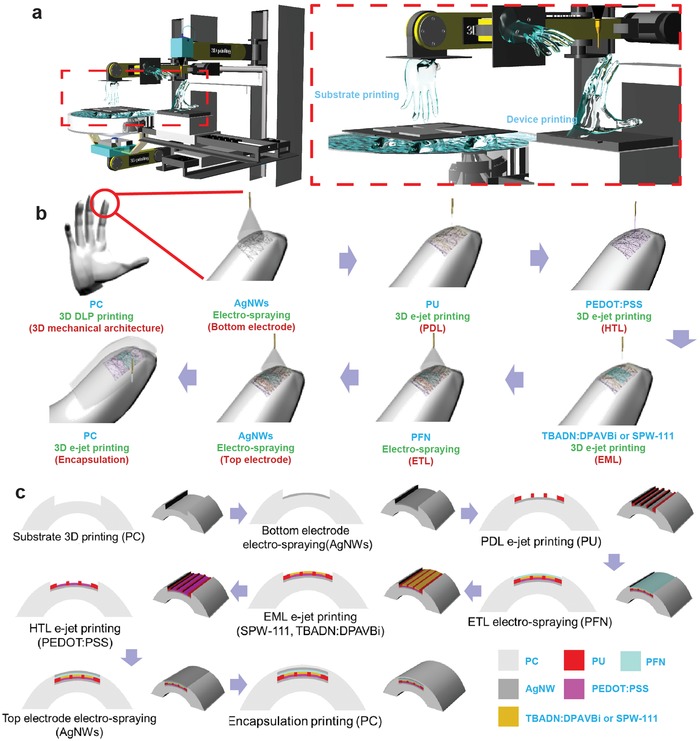
Schematic illustration of the hybrid 3D printing system and fabrication. a) Schematic illustration of the hybrid 3D printing system. b,c) Schematic illustration of the overall printing process for transparent 3D OLED devices.

For the DLP printing, the exposure of a photocurable PC resin (WaterClear Ultra 10122, Somos) to a laser at the wavelength of 405 nm for 10 s can pattern a 2D layer of PC, and then its layer‐by‐layer stacking enables the formation of transparent 3D architectures. The e‐jet printing system consists of a metal‐coated nozzle connected to a syringe, a computer‐controlled power supply, a pneumatic pressure controller, and the translational stage, which was controlled with five‐axis movements, i.e., three orthogonal directions and two tilting axes. First, the metal‐coated nozzles were prepared using a pipette puller to make glass capillaries with inner diameters (i.d.s) of 1–10 µm (Figure S1b,c, Supporting Information). Then, a thin Cu film (thickness: ≈100 nm) was evaporated to the capillary with a Cr adhesive layer (≈10 nm) before dipping this Cu‐coated capillary into a 1H,1H,2H,2H‐perfluorodecane‐1‐thiol solution (0.01 wt% in dimethylformamide) for 30 min to obtain a self‐assembled hydrophobic layer in order to prevent the ink from wetting the sidewall of the nozzle during the e‐jet printing process. Then, this nozzle was mounted to a syringe that was connected to a power supply. The 3D architecture that was preprinted using DLP was placed on the five‐axis stage for e‐jet printing, and the gap between the tip of a nozzle and the surface of this architecture was controlled to be ≈50 µm. The pneumatic pump delivered an ink from the syringe to the tip of the nozzle to form a pendant ink meniscus. When a voltage, i.e., an electric field, was applied to the nozzle, mobile charges inside the ink accumulated near the surface of the meniscus making the meniscus a conical shape, which is known as a Taylor cone.^41^, ^42^, ^43^ At sufficiently high electric fields (DC bias in the range of 100–600 V or AC bias in the range of 100–500 V, which had frequency ranges of 100–200 Hz, depending on the property of the ink and the size of the nozzle), a continuous jet was ejected or electro‐sprayed from the meniscus when the electrostatic force exceeded the counteracting capillary force.^41^, ^42^, ^43^ Then, the translation movements of the stage along the coordinate axes can result in 3D patterns. The uses of fine nozzles with small inner diameters (i.d. ≤ 10 µm) and relatively volatile solvents facilitated the rapid drying of the ink droplet printed on the surface of the PC (without additional heating), which can retard its wetting on the PC layer to form complex 3D features with high resolutions via the droplet‐by‐droplet assembly at ambient conditions.^44^ **Figure** **2**a–c shows examples of the optical micrographs of the e‐jet‐printed lines using PEDOT:PSS, SPW‐111, or PU with a 2 µm diameter nozzle. Figure 2d shows cross‐sectional profiles of the e‐jet‐printed patterns in Figure 2a–c. The HTL material (PEDOT:PSS), EML material (SPW‐111), and the PDL (PU) were printed with line widths of 2.8, 2.6, and 4.1 µm, respectively. This good printing resolution was essential for achieving higher integrations for miniaturized devices and better resolution displays. Figure 2e–g shows that overlapping the adjacent lines (with no gap) can print areal patterns with complex geometries instead of simple lines, such as an array of rectangles (Figure 2e, ink: PEDOT:PSS), layer‐by‐layer assembly of square patterns (Figure 2f, ink: SPW‐111), and a swan pattern (Figure 2g, ink: PU).

**Figure 2:**
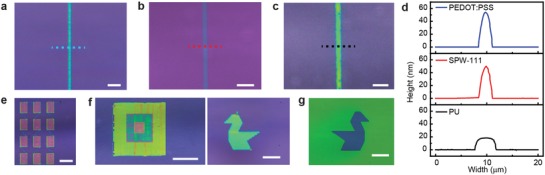
3D e‐jet printing of various materials and patterns. a–c) Optical micrographs of 3D e‐jet‐printed single line patterns of PEDOT:PSS, SPW‐111, and PU, respectively; the scale bars are 10 µm. d) Cross‐sectional profile of the printed single line along the indicated lines in (a)–(c). e) Optical micrograph of the 3D e‐jet‐printed PEDOT:PSS rectangular array. Scale bars are 100 µm. f) Optical micrographs of the 3D e‐jet‐printed SPW‐111 stacking, rectangular (left) and swan (right). Scale bars are 100 and 40 µm, respectively. g) Optical micrograph of 3D e‐jet‐printed PU intaglio swan pattern. Scale bar is 40 µm.

In practice, active materials need to be printed into the OLED pixels of the display for freeform architectures. Accordingly, after forming random networks of AgNWs as a transparent electrode using electrospray on the DLP‐preprinted PC surface, we printed the dielectric PDL pattern of PU using an e‐jet. This PDL pattern was additionally exposed to UV light (wavelength: 365 nm) for 6 s for photocuring after printing, and this cured PDL can eliminate the current leakage through unprinted regions between the anode and the cathode. Then, all of the openings inside the PDL pattern were filled with layers of PEDOT:PSS (HTL), SPW‐111 (EML), and PFN (ETL) in sequence using e‐jet printing to form an OLED pixel. Table S1 in the Supporting Information summarizes the experimental parameters for the e‐jet printing or electrospraying of these functional layers. **Figure** **3**a shows an optical micrograph of these OLED pixels and PDL patterns with the opening dimensions of 20 µm × 80 µm. These layers of HTL, EML, and ETL intentionally were printed with slightly larger dimensions than the actual sizes of the pixels to guarantee that the entire pixel area was filled with these active layers. Although all layers of these functional materials, as well as PDL, were printed using an e‐jet printer, the high‐resolution patterning of these pixels approached the subpixel sizes of the high‐definition, flat‐panel OLED displays that were fabricated recently using a conventional evaporation method with fine metal masks instead of printing. The surface morphology and the uniformity of the thicknesses of these active layers also were important for reliable OLED operations. As shown in Figure S2 in the Supporting Information, the 3D‐printed PC surface is smooth, and the surface roughness of the e‐jet printed layers was compared with the spin‐coated cases (Table S2, Supporting Information). 3D e‐jet printing system can produce smooth surfaces, comparable to the spun layers. Figure 3b–e shows atomic force microscopy (AFM) images of the red‐dashed area in Figure 3a, and Figure 3f shows cross‐sectional profiles of these e‐jet‐printed layers along the lines indicated in Figure 3b–e. Clearly stepped edges of PDL were observed after defining the pixel via e‐jet printing of PU, and Figure 3f indicates that e‐jet printing can provide good flatness of these active layers. Random networks of AgNWs covered the entire top surface of both ETL and PDL as a transparent electrode using electrospray, and the cross‐sectional images of this sample were obtained from the focused ion‐beam scanning electron microscopy (FIB‐SEM) images (Figure 3g). No abrupt change was observed in the thicknesses of any of the layers over the entire pixel area without any vacancies or defects.

**Figure 3:**
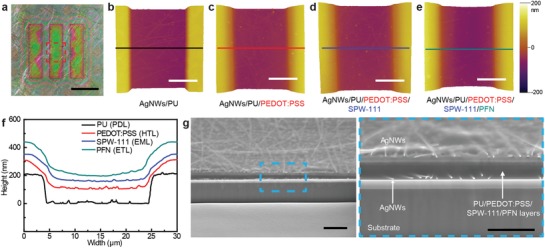
Cross‐sectional profile of a 3D e‐jet‐printed OLED. a) Optical micrograph of the 3D‐printed transparent OLED pixels. Scale bar is 40 µm. AFM images of the pixel area after each fabrication step of b) pixel defining, c) PEDOT:PSS, d) SPW‐111, and e) PFN 3D printing. Scale bars, 10 µm. f) Cross‐sectional profile of the pixel according to the AFM images in (b)–(e). g) FIB‐SEM images of 3D e‐jet‐printed transparent OLEDs. Right FIB‐SEM image is a magnified view of the blue box in the left side of the FIB‐SEM image. Scale bars are 1 µm (left) and 500 nm (right).

The resulting OLED pixels can be encapsulated by printing a transparent PC layer using e‐jet additionally, and **Figure** **4**a shows the schematic layout of the OLED device on a DLP‐preprinted 3D architecture. Figure 4b shows optical micrographs of the electroluminescence (EL) emissions from the printed OLED pixels using two different emission materials (EML), i.e., SPW‐111 (for the emission of white light) and TBADN:DPAVBi (for the emission of blue light). These images clearly show the defined light‐emitting pixels with distinct colors. The planar structure is required to measure the exact characteristics of the e‐jet‐printed OLEDs, because nonplanar surfaces (3D features) can distort the direction of light emission or vary the distance between the detector and the light source. Thus, a planar surface was printed using the DLP printer and then the OLEDs were printed using e‐jet on the preprinted planar surface. The plot of current density versus voltage in Figure 4c exhibits the stable operation of the printed OLED with a turn‐on voltage of 3 V for TBADN:DPAVBi (or 3.5 V for SPW‐111). As shown in the luminance versus voltage curves (Figure 4d), the luminances that were achievable at 12 V for the TBADN:DPAVBi sample and the SPW‐111 sample were about 1360 and 1235 cd m^−2^, respectively. Also, the performance of e‐jet‐printed or spin‐coated OLEDs fabricated using two different emission materials (TBADN:DPAVBi and SPW‐111) was compared. These samples (e‐jet‐printed or spin‐coated OLEDs) exhibited similar characteristics of the turn‐on voltage, luminance at 11 V, and maximum current efficiency, as shown in Figure S3 and Table S3 in the Supporting Information. Depending on the OLED materials, the maximum current efficiencies can appear at higher current densities, rather than lower current densities.^45^, ^46^ For example, the maximum current efficiency of the OLED using SPW‐111 appears at low current density, but the maximum current efficiency of the other OLED using TBADN:DPAVBi appears at high current density. We also compared the properties of the e‐jet‐printed OLED with the properties that have been reported in previous papers. As shown in Table S4 in the Supporting Information, the properties of the e‐jet‐printed OLED were comparable to the properties in previous results.^47^, ^48^, ^49^, ^50^ As shown in Figures S3 and S4 in the Supporting Information, when a reverse bias of 3 V was applied, the reverse currents (at 3 V reverse bias) of the OLEDs were 0.0052 mA cm^−2^ (EML: TBADN:DPAVBi) and 0.0025 mA cm^−2^ (EML: SPW‐111). In addition, their lifetimes were measured by operating these OLEDs at the initial luminance of 1000 cd m^−2^ at 25 °C with 45% relative humidity. The OLEDs were operated continuously, and their luminance was measured every 30 s. Then the lifetimes (*L*_70_, time for luminance to decline by 70% in air) of these OLEDs were measured. The *L*_70_ values of the OLEDs that were exposed to air after the devices were fabricated (without encapsulation) were only 0.7 h (EML: SPW‐111) and 1.2 h (EML: TBADN:DPAVBi). However, the *L*_70_ of the OLEDs increased to 6.5 h (EML: SPW‐111) and 7.5 h (EML: TBADN:DPAVBi) when they were encapsulated using a PC layer, as shown in Figure S5 in the Supporting Information. In the case of the nonencapsulated OLEDs, the top layer is the random networks of AgNWs with open spaces. Therefore, oxygen and water vapor can penetrate through these open spaces to degrade the nonencapsulated OLEDs. In the case of the encapsulated OLEDs, the PC encapsulation layer can protect the top OLED surface to retard the damage by oxygen and water vapor. In addition, the *L*_70_ values of the five devices were measured to compare the reliabilities of the spin‐coated OLEDs and the e‐jet‐printed OLEDs (Table S5, Supporting Information). Also, the processing steps of this hybrid 3D printing system require no additional thermal drying/annealing steps immediately after the materials were printed because our e‐jet system uses fine nozzles to facilitate the ejection of small droplets, which are dried very quickly during their flight.^44^, ^51^ However, additional thermal treatments of the fully fabricated OLEDs can improve the device performances further by enhancing the diffusivity of carriers.^52^, ^53^ As shown in Figure S4 in the Supporting Information, the properties (turn‐on voltage, luminance at 11 V, and maximum current efficiency) of the e‐jet‐printed OLEDs with or without the thermal drying process (130 °C for 10 min) were not significantly different. As an example, Movie S1 in the Supporting Information presents the on/off operation of the printed OLED device with blue light emission using TBADN:DPAVBi. In this movie, the gradual increase in the DC bias applied to this OLED sample increased the luminance. Also, Figure 4e shows the normalized EL spectra of the e‐jet‐printed OLED, verifying the bluish emission for TBADN:DPAVBi and the whitish emission for SPW‐111. At 550 nm, the transmittances of the OLED pixels based on TBADN:DPAVBi and SPW‐111 were 70.1% and 75.3%, respectively (Figure 4f). For this measurement, the transparent OLED size larger than the laser spot (diameter: 2 mm) was printed, and these samples were detected using UV‐vis–NIR spectroscopy (Cary 5000 UV‐Vis‐NIR, Agilent).

**Figure 4:**
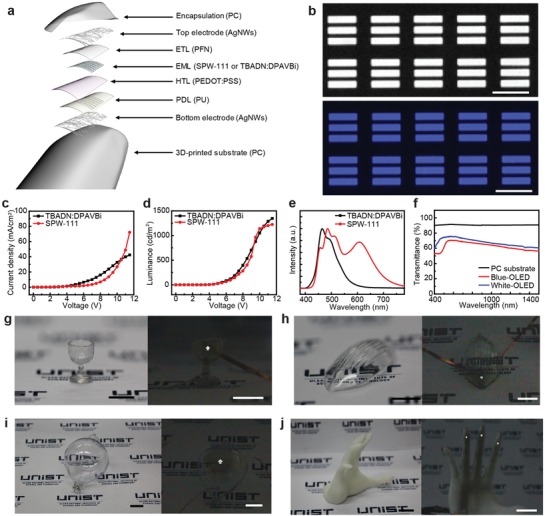
Transparent OLEDs on various 3D architectures. a) Schematic illustration of the transparent OLED structure. b) Optical micrographs of EL emission from the fabricated OLEDs with TBADN:DPAVBi (top) and Merck White (bottom). Scale bars, 100 µm. c) Current density versus voltage curves of the 3D e‐jet‐printed OLED device with TBADN:DPAVBi (black) and Merck White (red). d) Luminance versus voltage curves of the 3D e‐jet‐printed OLED device with TBADN:DPAVBi (black) and Merck White (red). e) Normalized EL spectrum of the TBADN:DPAVBi (black) and Merck White (red). f) Optical transmittance spectrum of the 3D‐printed PC substrate, TBADN:DPAVBi, and SPW‐111‐based transparent OLEDs. g–j) Photographs of the 3D‐printed transparent OLEDs in turn‐off (left) and turn‐on (right) condition: g) cup of glass, scale bar, 5 mm. h) lotus leaf dish, i) round shape lamp, and j) human hand. Scale bars, 1 cm.

A key advantage of this hybrid 3D printing system was its ability to print entire components of the OLED devices as well as arbitrary‐shaped plastic frames by embedding these optoelectronic devices inside 3D architectures as freeform optoelectronics. For this demonstration, Figure 4g–j shows photographs of the OLED devices embedded inside diverse plastic sculptures, such as a transparent cup (Figure 4g), a lotus leaf dish (Figure 4h), a rounded lamp (Figure 4i), and a human hand (Figure 4j). Diverse geometries of the OLED patterns in Figure 4g–j consist of multiple pixels with each dimension of 20 µm × 80 µm (Figure S6, Supporting Information). To print the ink at the exact position, the gap between the tip of a nozzle and the surface of this architecture was controlled to be ≈50 µm. The topology profile of the 3D‐printed freeform surface was input to the e‐jet printer controller. The five‐axis system enables the *z*‐axis translation with *xy‐*plane tilting. This can maintain a constant stand‐off distance between the nozzle tip and substrate surface during printing even for nonplanar surfaces (Figure S7, Supporting Information). Both transparent PC and opaque PLA were used here, and the turn‐on (or turn‐off) conditions of the OLED pixels are shown in the left (or right) insets of Figure 4g–j. Movie S2 in the Supporting Information presents the real‐time operations of these samples. Diverse geometries of the pixel patterns (e.g., circles or arrows) as well as 3D plastic frames can be printed, and these demonstrations represent substantial progress toward next‐generation, freeform optoelectronics.

As another example of an application, **Figure** **5** shows the hybrid 3D printing of a transparent, eyeglass‐type display for a wireless, augmented reality system. Transparent PC eyeglasses with OLED pixels were printed using e‐jet before interconnecting them with a battery, microcontroller unit (MCU), and Bluetooth module to control the intensity of the OLED emission using a smartphone wirelessly (Figure 5a). Figure S8 in the Supporting Information shows the corresponding circuit diagram. The digital output signal from MCU applies a voltage to the LED driver, which was directly connected to the OLED pixels, and it can turn these pixels on or off. After loading a custom‐designed program on MCU, MCU was integrated with the Bluetooth module to control the level of the operating current.^54^ This electric current controls the light intensity emitted from OLED pixels using a smartphone wirelessly. The wireless communication capability of this eyeglass‐type display with mobile devices can provide the automated machine learning function (via smartphone software) for augmented reality applications. In our smartphone program, users can control the intensity of the emitted light with 255 steps, as shown in Figure 5b. The input voltage of 3.7 V is amplified to the output voltage of 8 V through MCU, and this relatively low bias is enough to operate this OLED display with a current less than 50 mA. Figure 5c shows photographs of this 3D‐printed, eyeglass‐type display. The left inset shows the “turn‐off” condition of this display, which was placed in front of a paper to demonstrate its transparent properties, and the right inset presents the wireless operation (turn‐on) of this display. In addition, Movie S3 in the Supporting Information demonstrates a human subject wearing this eyeglass‐type display that allows real‐time, wireless control of the light intensity from OLED pixels using a mobile phone.

**Figure 5:**
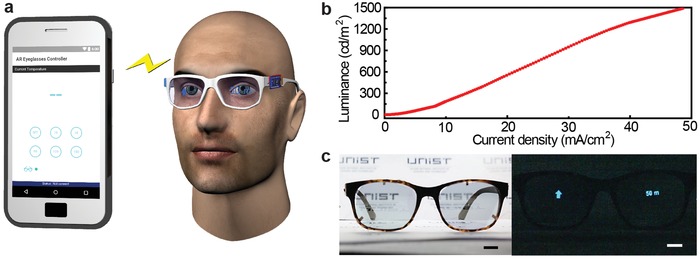
Examples of applications of the 3D‐printed transparent OLEDs as augmented reality eyeglasses. a) Schematic illustration of the 3D‐printed augmented reality eyeglasses. b) Luminance versus current density curve of transparent OLED in augmented reality eyeglasses. c) Photographs of demonstrated augmented reality eyeglasses in turn‐off (left) and turn‐on (right) condition. Scale bars, 5 mm.

In conclusion, the work presented here demonstrates a hybrid 3D printing system that combines DLP and e‐jet for the production of freeform and transparent optoelectronic devices in ambient air. The 3D architectures of transparent or opaque plastics with arbitrary shapes are printed using DLP, and then all active materials of the OLED displays are printed directly on the 3D surfaces using an e‐jet with high resolutions and with no additional thermal annealing steps. Although previous works have been limited to printing only a few components of OLED devices on flat substrates,^55^, ^56^ our approach allows the printing of all components of the device and the mechanical frames in ambient air. This allows transparent OLED pixels to be embedded inside the freeform 3D architectures at desired locations. Also, the integration of these transparent and freeform displays with wireless communication circuits demonstrates their substantial progress for use as augmented reality systems. In fact, we expect that the printing speed can be improved further by multiple‐nozzle implementations of the e‐jet that would be conceptually similar to those used in conventional inkjet printheads. Also, the development of new 3D‐printable encapsulation materials with lower water and oxygen permeability can increase the OLED lifetime further. These process improvements, together with exploration of applications in biotechnology and other areas, represent promising areas for future work.

## Experimental Section

Detailed descriptions of materials and fabrication processes can be found in the Supporting Information.

## Conflict of Interest

The authors declare no conflict of interest.

## Supporting information

Supplementary

Supplementary

Supplementary

Supplementary
