# Supplementary material for: High‐Resolution 3D Printing of Freeform, Transparent Displays in Ambient Air
Source: Adv Sci (Weinh). 2019 Oct 4;6(23):1901603. doi: 10.1002/advs.201901603 (PMC6891910; doi:10.1002/advs.201901603)
Supplement: Supplementary file 1 — Supplementary [file ADVS-6-1901603-s002.pdf]

Copyright WILEY-VCH Verlag GmbH & Co. KGaA, 69469 Weinheim, Germany, 2019.

Supporting Information

for *Adv. Sci.,* DOI: 10.1002/advs.201901603

High-Resolution 3D Printing of Freeform, Transparent Displays in Ambient Air

*Hyeon Seok An, Young-Geun Park, Kukjoo Kim, Yun Seok Nam, Myoung Hoon Song, and Jang-Ung Park**

Copyright WILEY-VCH Verlag GmbH & Co. KGaA, 69469 Weinheim, Germany, 2019.

Supporting Information

High-Resolution 3D Printing of Freeform, Transparent Displays in Ambient Air

*Hyeon Seok An, Young-Geun Park, Kukjoo Kim, Yun Seok Nam, Myoung Hoon Song, Jang-Ung Park**

Dr. H. S. An, Y.-G. Park, Prof. J.-U. Park
Nano Science Technology Institute, Department of Materials Science and Engineering, Yonsei University, Seoul, 03722, Republic of Korea
Dr. H. S. An, Y.-G. Park, J.-U. Park
Center for Nanomedicine, Institute for Basic Science (IBS), Yonsei-IBS Institute, Seoul, 03722, Republic of Korea
Dr. K. Kim
Electronics and Telecommunications Research Institute (ETRI), Daejeon Metropolitan City, 34129, Republic of Korea
Y. S. Nam, Prof. M. H. Song
School of Materials Science and Engineering, Ulsan National Institute of Science and Technology (UNIST), Ulsan Metropolitan City, 44919, Republic of Korea

E-mail: jang-ung@yonsei.ac.kr

Keywords: 3D printing, printable electronics, optoelectronics, transparent display

This PDF file includes:

Methods

Supporting Figures S1-S8

Supporting Table S1-S5

Captions of Supporting Movies S1-S3

Methods

Preparation of nozzles

A glass pipette (World Precision Instruments) whose outer diameter is 1.0 mm and inner diameter is 0.5 mm was pulled to prepare a nozzle whose inner diameter is 1-5 μm with a pipette puller (Sutter P-1000). Cr (10 nm)/Cu (100 nm) layer was coated onto pre-pulled glass pipettes using a thermal evaporator. The tip of the metal-coated nozzle was dipped into 1H,1H,2H,2H-per-fluorodecane-1-thiol (Sigma-Aldrich) solution (0.1 wt% in dimethylformamide) for 30 min to make a hydrophobic self-assembled layer on the surface of the nozzle tip.

Ink preparation

Hole transport layer: Poly(3,4-ethylenedioxythiophene)/poly(styrenesulfonate) (PEDOT:PSS) (Clevios AI 4083, Heraeus, Germany) was mixed with deionized water (18.2 MΩ·cm at 25°C) and ethylene glycol in a weight ratio of 5:7:2.

Emissive layers: Super yellow (Merck Co., Darmstadt, Germany) solution dissolved in chlorobenzene (6.2 mg/ml) for the emissive layer

Pixel defining layer: NOA (Norland Optical Adhesive 74, Norland Products, Inc., USA) was ultraviolet-ozone treated to have a viscosity optimized for printing.

Anode and Cathode materials: The AgNWs (Nanopyxis, Korea) was dispersed in IPA to a concentration of 0.5 wt%.

Fabrication of OLEDs

The AgNW transparent electrode was electro-sprayed on the 3D-printed PC substrate. The PEDOT:PSS was filtered by a hydrophilic filter with 450-nm pores and 3D printed on the 3D-printed substrate. After the PEDOT:PSS was dried, the emission layer (TBADN:DPAVBi or

SPW-111) was printed using the 3D e-jet printing method. PFN was electro-sprayed continuously as the ETL. The AgNWs were electro-sprayed on top of the 3D-printed OLEDs as a top electrode. These printed OLED pixel patterns can be dried at 130 ºC for 10 min to make sure the complete drying of solvents. Last, the PC layer was 3D printed on the transparent OLEDs as an encapsulation layer.

3D E-jet printing

The temperature (20 ºC) and relative humidity (10%) were constantly maintained during the printing process. The 3D-printed substrate was positioned on a high-resolution translation stage below the nozzle which was preliminarily coated by a thin metal film to generate an electric field within the stage. Nozzles with internal diameters of 1 or 2 μm were used. The stage translated at a speed of 300 μm/s during the printing process. The ink was supplied by pneumatic pressure (0.1 psi) from the syringe to the nozzle tip. The distance between the nozzle and the substrate was fixed at 15 μm and the DC voltage was controlled in the range of 100-400 V. Table S1 summarizes the printing conditions for this work.

OLED characterization

J−V−L characteristics of OLEDs was carried out using spectroradiometer (CS-2000, Konica Minolta Co.) with source measurement unit (Keithley 2400, Keithley).

Optical Characterization

The Raman spectra were obtained with a WITec CRM200 Raman system with a 532 nm laser as the excitation source. The optical transmittance of films was measured using UV-Vis-NIR spectroscopy (Cary 5000 UV-Vis-NIR, Agilent) with the transmittance of the substrate as a baseline.

Supporting Figures

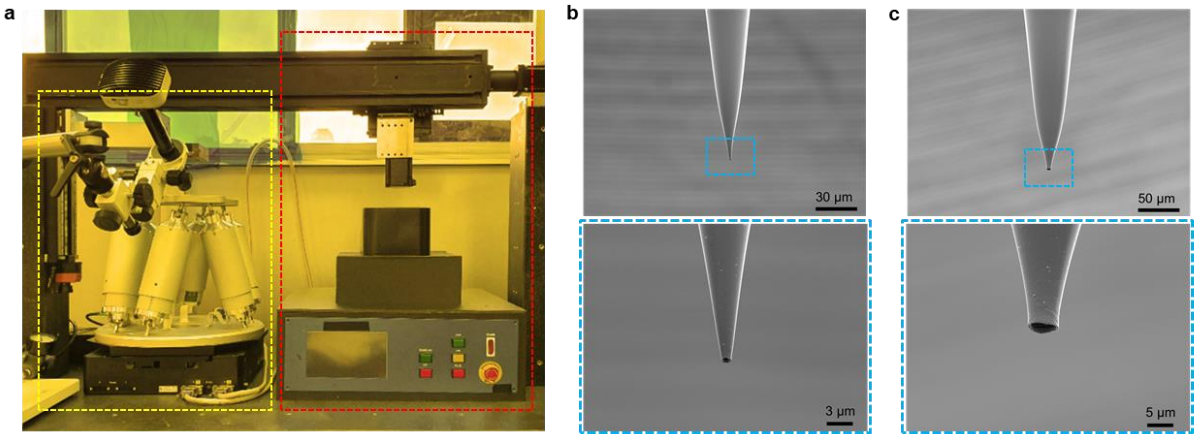

Figure S1. a) Photograph of the printer setup. The yellow- and red-dashed rectangles indicate the e-jet printing and DLP printing system, respectively. **b**) SEM images of the e-jet nozzle with 1 µm of its inner diameter. **c**) SEM images of the e-jet nozzle with 5 µm of its inner diameter. Blue-dashed rectangles present the magnified view of the nozzle tip.

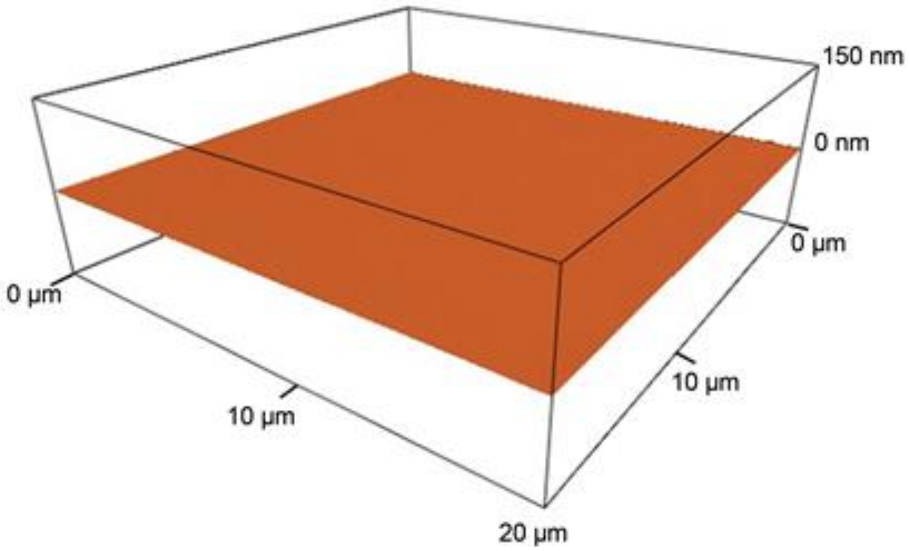

Figure S2. AFM image of 3D-printed PC architecture.

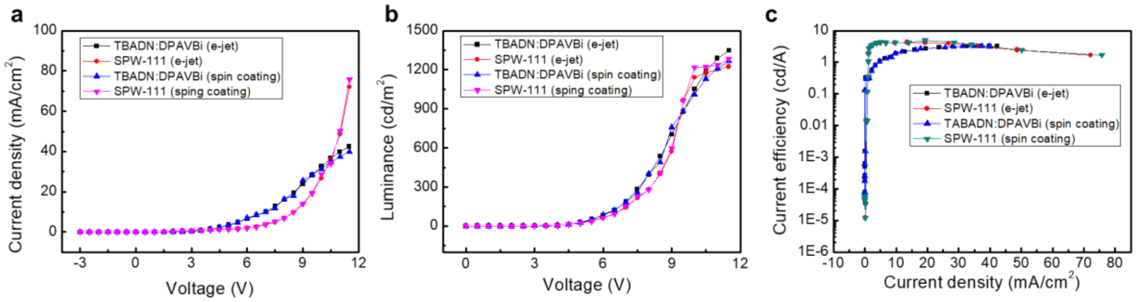

Figure S3. The properties of e-jet printed or spin-coated OLEDs fabricated using two different emission materials (TBADN:DPAVBi and SPW-111) **a)** Comparison of current density *vs.* voltage. **b)** Comparison of luminance *vs.* voltage. **c)** Comparison of current efficiency *vs.* current density.

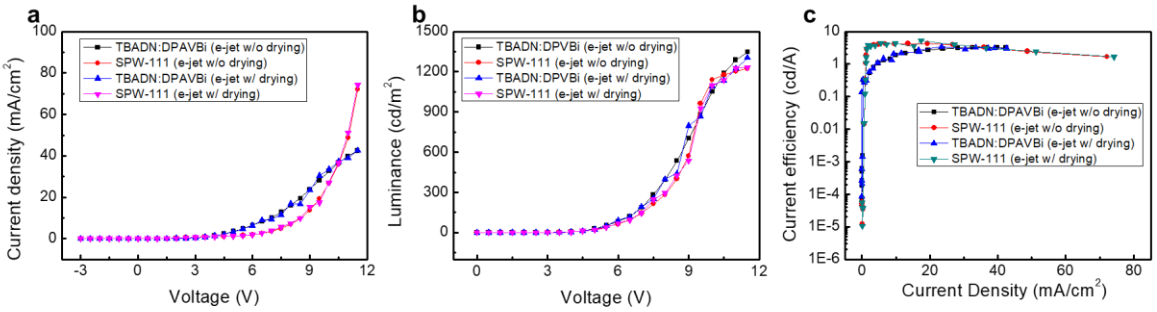

Figure S4. Properties of the e-jet printed OLEDs with or without the thermal drying (130 ^{o}C for 10 minutes). **a)** Comparison of current density *vs.* voltage. **b)** Comparison of luminance *vs.* voltage. **c)** Comparison of current efficiency *vs.* current density.

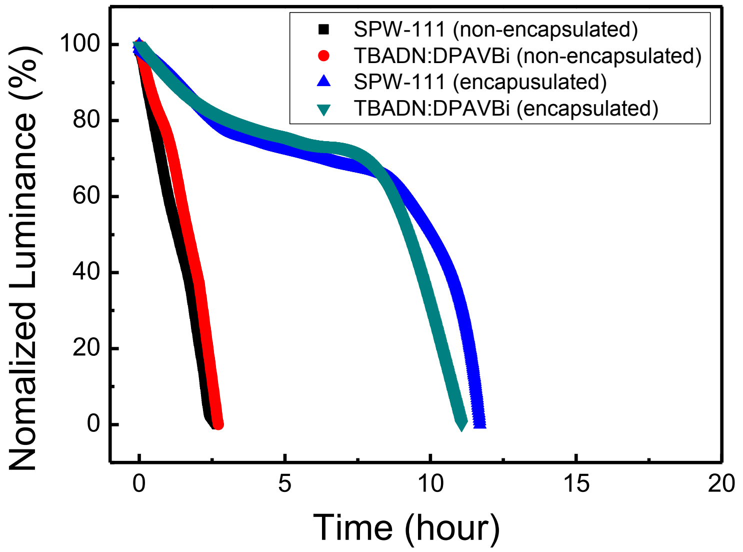

Figure S5. The luminance change of the OLED samples with or without the encapsulation PC layer.

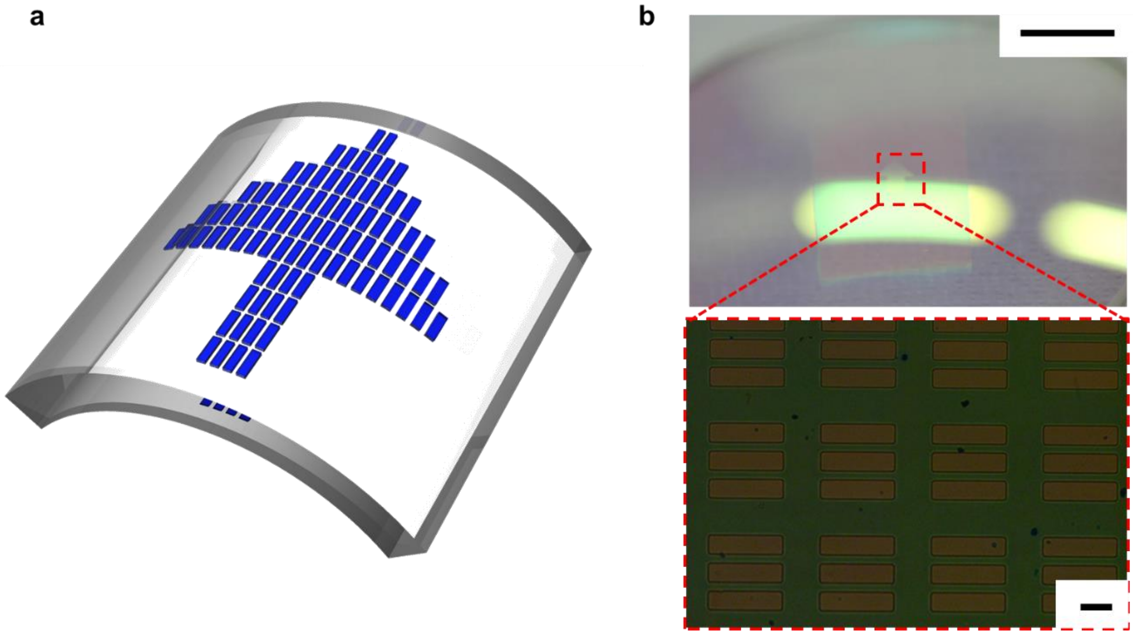

Figure S6. Schematic illustration, photograph, and optical micrograph of the 3D printed OLEDs. a) schematic illustration of the nonplanar OLED pattern. b) Photograph (top) and optical micrograph (bottom) of OLEDs. Scale bars are 1 cm (top) and 50 µm (bottom).

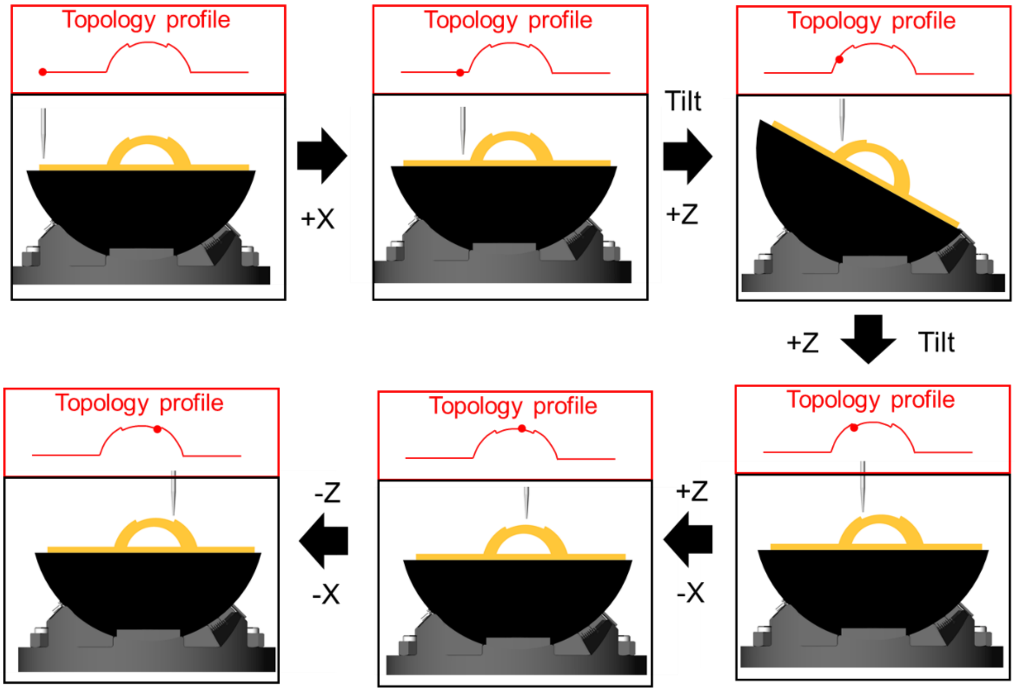

Figure S7. Control of the stand-off height and position between the nozzle and nonplanar surface. The 5-axis system enables the z-axis translation with *xy*-plane tilting, which can maintain a constant stand-off distance and control position between the nozzle tip and substrate surface during printing even for nonplanar surfaces.

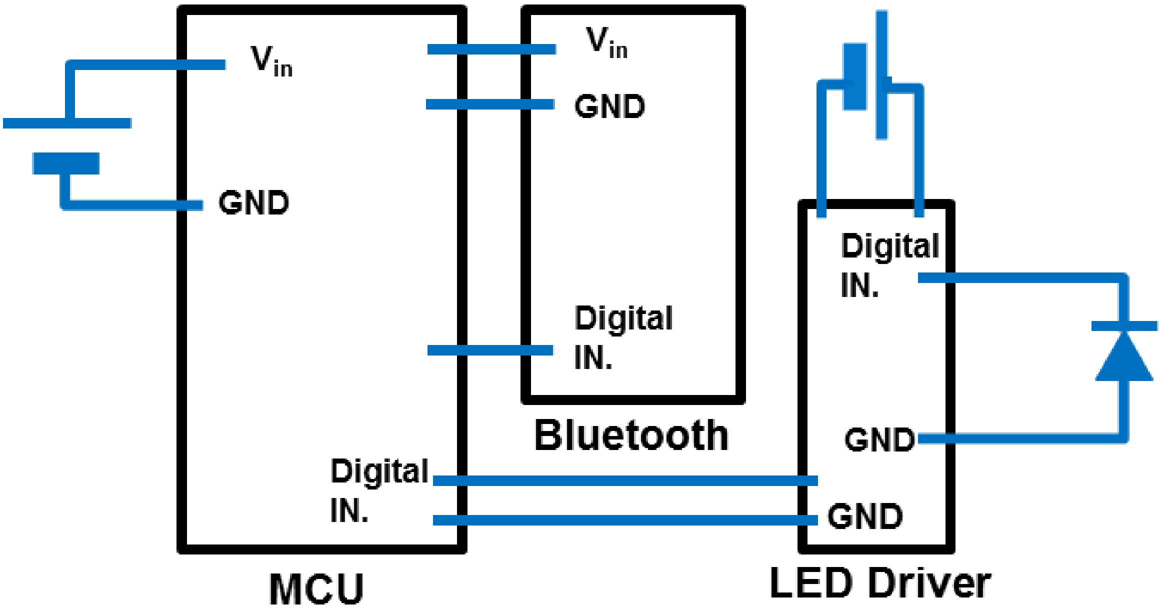

Figure S8. Corresponding circuit diagram of the Bluetooth module

Supporting Tables

Layer	Nozzle diameter	Nozzle-substrate distance	Voltage	Pneumatic pressure	Printing speed
Bottom electrode (AgNWs)	200 μm	10 cm	1.2 kV	35 psi	-
PDL (PU)	2 μm	10~30 μm	100 ~ 400 V	0.2 psi	0.8 mm/s
HTL (PEDOT:PSS)	2 μm	10~30 μm	100 ~ 400 V	1 psi	0.8 mm/s
EML (SPW-111, TBADN:DPAVBi)	2 μm	10~30 μm	100 ~ 400 V	0.5 psi	0.8 mm/s
ETL (PFN)	200 μm	3 cm	600 V	50 psi	-
Top electrode (AgNWs)	200 μm	10 cm	1.2 kV	35 psi	-

Table S1. Summarized printing conditions.

	E-jet printing	Spin coating
PEDOT:PSS (HTL)	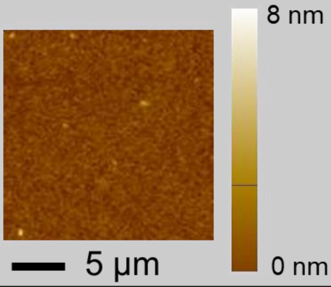	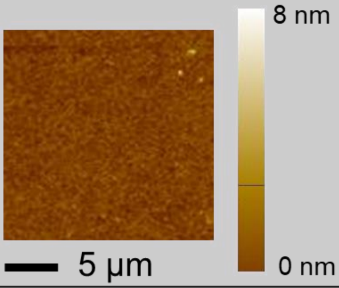
R.M.S. roughness (nm)	1.412	1.302
TBADN:DPAVBi (EML)	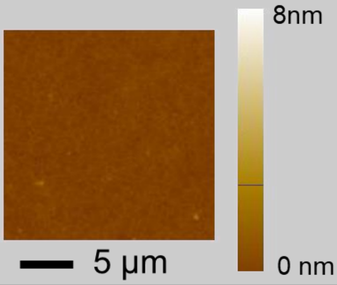	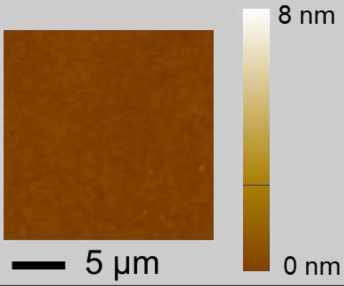
R.M.S. roughness (nm)	0.615	0.587
SPW-111 (EML)	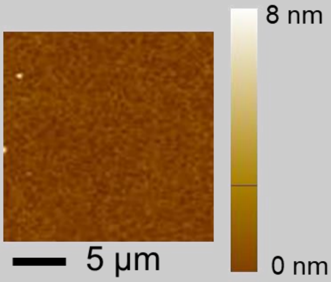	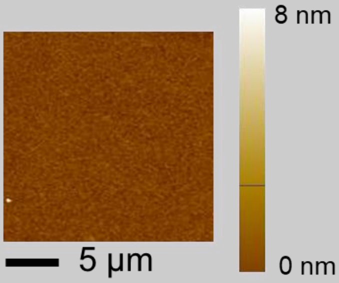
R.M.S. roughness (nm)	0.918	1.052
PFN (ETL)	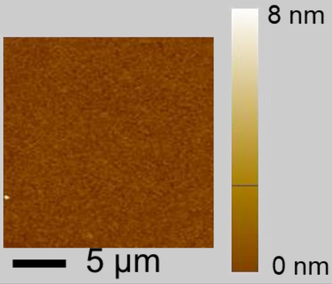	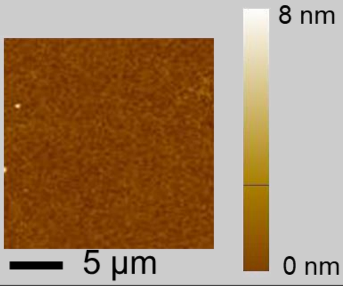
R.M.S. roughness (nm)	1.382	1.352

Table S2. AFM images and RMS roughness of the e-jet printed or spin-coated layers. Here the PC layer was used as the substrate.

Emission material	TBADN:DPAVBi			SPW-111		
	Turn-on voltage (V)	Luminance @ 11 V (cd/m^2)	Maximum current efficiency (at listed voltage) (cd/A)	Turn-on voltage (V)	Luminance @ 11 V (cd/m^2)	Maximum current efficiency (at listed voltage) (cd/A)
E-jet printed OLED	3 ± 0.12	1,288 ± 12	3.25 ± 0.11 (10.5 V)	3.5 ± 0.13	1,201 ± 15	4.23 ± 0.15 (9.5 V)
Spin-coated OLED	3 ± 0.11	1,211 ± 15	3.25 ± 0.1 (10.5 V)	3.5 ± 0.14	1,238 ± 13	4.31 ± 0.12 (9.5 V)

Table S3. Properties of e-jet printed or spin-coated OLEDs fabricated using two different emission materials (TBADN:DPAVBi or SPW-111).

Emission material		Blue OLED					White OLED				
		Turn-on voltage (V)	Maximum luminance (at listed voltage) (cd/m^2)		Maximum current efficiency (at listed voltage) (cd/A)		Turn-on voltage (V)	Maximum luminance (at listed voltage) (cd/m^2)		Maximum current efficiency (at listed voltage) (cd/A)	
E-jet printed OLED<br>Total		3 ± 0.12	2,561 ± 21 (11 V)		6.49 ± 0.21 (10.5 V)		3.5 ± 0.13	2,405 ± 29 (11 V)		8.45 ± 0.29 (9.5 V)	
Top	Bottom		1,282 ± 11 (11 V)	1,279 ± 10 (11 V)	3.25 ± 0.11 (10.5 V)	3.24 ± 0.09 (10.5 V)		1,201 ± 15 (11 V)	1,198 ± 14 (11 V)	4.23 ± 0.15 (9.5 V)	4.22 ± 0.14 (9.5 V)
Ref 48 (transparent)<br>Total		3.3	2,700 (6.5 V)		2.5 (4.8 V)		-	-		-	
Top	Bottom		1,200 (6.5 V)	1,500 (6.5 V)	1.1 (4.8 V)	1.4 (4.8 V)					
Ref 49 (opaque)		-	-		-		6	3093 (13 V)		0.295 (11 V)	
Ref 50 (opaque)		-	-		-		2.2	5,500 (4.8 V)		0.46 (4.8 V)	
Ref 51 (transparent)<br>Total		-	-		-		7	2,190 (21 V)		4.2 (17 V)	
Top	Bottom							1,090 (21 V)	1,100 (21 V)	2.1 (18 V)	2.1 (16.5 V)

Table S4. Properties of e-jet printed OLED and their comparison with other OLED cases using the same EML materials (TBADN:DPAVBi or SPW-111).

	L_{70} (time for luminance to decline by 70% in air)
TBADN:DPAVBi (e-jet)	6.5 ± 0.32
SPW-111 (e-jet)	7.5 ± 0.35
TBADN:DPAVBi (spin coating)	6.6 ± 0.28
SPW-111 (spin coating)	7.8 ± 0.29

Table S5. Lifetime of the e-jet printed or spin-coated OLEDs.

Captions of Supporting Movie

Supporting Movie S1. The on/off operation of the printed OLED device with blue light emission using TBADN:DPAVBi.
Supporting Movie S2. The printed OLEDs with complex 3D architecture operation movie, such as cup of glass, lotus leaf dish, round-shape lamp and human hand.
Supporting Movie S3. The printed augmented reality eyeglasses operation movie using Bluetooth module and smartphone
